# Clinical variables provide the main predictive signal for early in-hospital rehabilitation triage after small-artery occlusion stroke: development and internal validation of an interpretable model

**DOI:** 10.3389/fneur.2026.1910602

**Published:** 2026-09-11

**Authors:** Yingqi Lu, Mei Shen, Xin Chen, Youli Jiang, Yue Wang, Qiuyang Qian, Qingshi Zhao, Wei Wang

**Affiliations:** 1 Department of Rehabilitation Medicine, People’s Hospital of Longhua, Shenzhen, China; 2 Department of Neurologic Medicine, People’s Hospital of Longhua, Shenzhen, China

**Keywords:** clinical prediction model, early in-hospital rehabilitation, interpretable machine learning, SHAP (SHapley Additive exPlanations), small artery occlusion stroke

## Abstract

**Background:**

Small artery occlusion stroke is often mild, but some patients need rehabilitation during acute hospitalization. We developed and internally validated an interpretable model to assist early in-hospital rehabilitation screening and triage and tested whether white matter hyperintensity burden and inflammatory-metabolic biomarkers improved prediction beyond routine clinical variables.

**Methods:**

Consecutive adults with acute ischemic stroke classified as small artery occlusion were identified from a single-centre stroke registry and electronic health records. Early rehabilitation need was assessed within 48 h after admission by trained rehabilitation physicians using a structured protocol. The prespecified primary model used clinical variables. Extended models added modified Fazekas score, inflammatory metabolic biomarkers, or both. Eight regression and machine learning algorithms were compared. Internal validation used stratified tenfold cross validation with nested fivefold tuning. Performance was assessed using discrimination, precision recall, calibration, and decision curve analysis.

**Results:**

Among 411 patients, 155 had early rehabilitation need (37.7%). The clinical-variable model performed best, with an area under the receiver operating characteristic curve of 0.682 and an area under the precision recall curve of 0.591. Adding modified Fazekas score, inflammatory metabolic biomarkers, or both did not improve performance. Logistic regression performed comparably to complex algorithms. In the admission fixed-landmark sensitivity analysis, the clinical-variable model yielded an AUROC of 0.623, which decreased to 0.507 after variables directly reflecting acute neurological deficits were excluded. Leading predictors reflected neurological severity, early functional impairment, and care pathway characteristics.

**Conclusion:**

Early rehabilitation need was common among patients with small-artery occlusion stroke during acute hospitalization. Routine clinical variables provided the dominant predictive information, whereas white matter hyperintensity burden and inflammatory-metabolic biomarkers added no stable incremental value.

## 1 Introduction

Small artery occlusion stroke is a major subtype of acute ischemic stroke and is closely related to perforating artery disease, lacunar infarction, and cerebral small vessel disease (1). It is usually characterised by small lesion volume, mild neurological deficits, and favourable short-term outcomes (2). Consequently, it is often regarded as mild stroke subtype in clinical practice (3). However, mild symptoms at presentation do not necessarily imply a low rehabilitation burden (4). Some patients still experience limb weakness, gait impairment, dysphagia, or impaired activities of daily living (5). Although these deficits are rarely life-threatening, they may hinder early mobilisation, complication prevention, and post-discharge recovery (6). Early functional assessment is therefore essential to detect rehabilitation needs that may otherwise be overlooked in this population.

Acute-phase rehabilitation assessment is a core component of stroke unit care (7). Early identification of rehabilitation needs supports timely in-hospital rehabilitation planning and continuity of post-discharge care (8). In small artery occlusion stroke, mild initial neurological deficits may lead clinicians to underestimate rehabilitation needs (9). Early rehabilitation need should therefore not be equated with actual rehabilitation service use (10). Rather, it represents a clinical triage indicator for further rehabilitation assessment and intervention (11).

Most existing stroke prediction studies focus on hard clinical endpoints: mortality, recurrence, neurological deterioration, and long-term functional outcomes (12). Early rehabilitation need during acute hospitalization, by contrast, remains vastly understudied, particularly among patients with small-artery occlusion stroke (13). White-matter hyperintensity burden and inflammatory-metabolic biomarkers are established correlates of post-stroke functional prognosis (14). It is still unclear whether they provide incremental predictive value over routine clinical variables for identifying early rehabilitation need (15). While regression and machine learning methods can integrate multi-domain predictors into a unified risk framework, rehabilitation triage demands more than strong discrimination. Interpretability and clinical accessibility are equally critical (16).

This study aimed to develop and internally validate an interpretable clinical variable model for early rehabilitation need in patients with small artery occlusion stroke using data from a single-centre stroke registry and electronic health records. It also assessed whether white matter hyperintensity burden and inflammatory metabolic biomarkers provided incremental predictive value beyond clinical variables. Multiple regression and machine learning algorithms were used for secondary benchmarking, and model discrimination, calibration, and clinical net benefit were systematically evaluated. SHapley Additive exPlanations (SHAP) were used to interpret variable contributions to model output. This analytical framework may support early rehabilitation risk stratification and inform more efficient use of rehabilitation resources in patients with small artery occlusion stroke.

## 2 Materials and methods

### 2.1 Ethics approval and study population

This was a single-centre retrospective prediction model study designed to develop and internally validate a model using data from the hospital stroke registry and electronic health records. The study was approved by the Ethics Committee of Shenzhen Longhua District People’s Hospital (approval number: 2024073-01). Because this study used retrospective data collected during routine clinical care and all data were anonymised before analysis, the Ethics Committee waived the requirement for informed consent.

Patients hospitalised with acute ischemic stroke from 2022 to 2023 were consecutively screened. Small artery occlusion was classified according to the Trial of Org 10172 in Acute Stroke Treatment (TOAST) classification criteria. The inclusion criteria were as follows: age of 18 years or older, diagnosis of acute ischemic stroke, classification as small artery occlusion, availability of early after admission clinical, imaging, and laboratory data for modeling, and documented early rehabilitation need assessment. The exclusion criteria were missing primary outcome data, substantial missingness in key baseline variables, uncertain temporal ordering between candidate predictors and outcome assessment, and severe non stroke conditions that could influence rehabilitation need assessment, including preexisting severe disability, severe musculoskeletal disease, end stage disease, or severe impairment of consciousness.

TOAST classification was used only for retrospective cohort selection and was not included as a model predictor. After completion of the etiological work-up, two senior neurologists assigned the final subtype based on clinical findings, magnetic resonance imaging (MRI) with diffusion-weighted imaging (DWI), vascular imaging using magnetic resonance angiography (MRA), and cardiac evaluation. Small-artery occlusion was defined as a compatible lacunar syndrome with an acute subcortical infarct and no competing mechanism, including ≥ 50% stenosis of the relevant parent artery, high-risk cardioembolism, or definite branch atheromatous disease. Acute ischemic stroke was defined as admission within 7 days of symptom onset or last known well. For wake-up or unknown-onset stroke, the last known well time was used as the reference.

A total of 417 patients with acute ischemic stroke classified as small artery occlusion were screened. Of these, six patients were excluded because early rehabilitation need assessment data were unavailable, leaving 411 patients in the final analysis.

### 2.2 Outcomes

The primary outcome was early rehabilitation need during acute hospitalization, defined as the need for further structured rehabilitation assessment within 48 h of admission. It did not indicate actual referral or rehabilitation treatment. The assessment covered neurological deficits, motor function, activities of daily living, and gait ability. No fixed numerical threshold was used; classification was based on overall clinical judgment across these domains. Each patient was independently assessed by two rehabilitation physicians with more than 5 years of experience. All assessors received standardized training and case-based calibration. Inter-rater agreement was good (Cohen’s κ = 0.81), and disagreements were resolved by a third senior physician. Assessors were not fully blinded to clinical information but were unaware of the study hypothesis, predictor grouping, and model analyses. They also had no access to post-assessment treatment or discharge outcomes. Patients requiring further structured rehabilitation assessment were coded as 1; others were coded as 0.

The intended users were stroke-unit clinicians involved in rehabilitation screening and referral. The model was intended for use within 48 h after admission to prioritize patients for formal rehabilitation assessment. A positive prediction indicated priority for assessment rather than automatic initiation of rehabilitation treatment. Candidate predictors were restricted to information available before the rehabilitation physician assessment. Variables recorded after the early rehabilitation need decision, treatment initiation, discharge evaluation, or follow-up were excluded. When timestamps were incomplete, clinical records were reviewed to confirm that candidate predictors preceded outcome assessment. Because early rehabilitation need partly reflects neurological severity and early functional status, national institutes of health stroke scale (NIHSS) score, modified rankin scale (mRS) score at 24 h after admission, and gait ability within 48 h were retained as clinically relevant early in-hospital predictors in the primary analysis. Their conceptual proximity to the outcome was explicitly evaluated in sensitivity analyses.

The secondary outcome was discharged functional dependence, defined as a modified Rankin Scale score of 2 or higher at discharge. The exploratory outcome was residual neurological deficit at discharge, defined as an NIHSS score of 3 or higher at discharge. The three outcomes were modelled and evaluated separately.

### 2.3 White matter hyperintensity assessment

White matter hyperintensity burden was assessed using the modified Fazekas score. Periventricular and deep white matter hyperintensities were each scored from 0 to 3, yielding a total score from 0 to 6. A higher score indicated more severe white matter hyperintensity burden. High Fazekas burden was defined as a modified Fazekas total score of 2 or higher.

Two radiologists independently scored the images and were blinded to early rehabilitation need and discharge outcomes. Inter-rater reliability for the modified Fazekas total score was assessed using the intraclass correlation coefficient (ICC), with an ICC of 0.87, indicating good agreement. Because imaging markers such as lacunes, cerebral microbleeds, and enlarged perivascular spaces were not systematically recorded in all patients, only the modified Fazekas score was used as an indicator of imaging burden. Therefore, overall cerebral small vessel disease burden was not defined.

### 2.4 Candidate predictors and predictor sets

Candidate predictors were restricted to variables available before outcome assessment. Clinical variables included demographic characteristics, vascular risk factors, early neurological and functional assessments, care pathway variables, vital signs, and onset-to-arrival time. Imaging variables included the modified Fazekas score. Inflammatory metabolic biomarkers included white blood cell count, neutrophil count, lymphocyte count, neutrophil-to-lymphocyte ratio, fibrinogen, fasting blood glucose, glycated haemoglobin, low-density lipoprotein cholesterol, serum uric acid, serum creatinine, blood urea nitrogen, and alanine aminotransferase.

The neutrophil-to-lymphocyte ratio was calculated as neutrophil count divided by lymphocyte count. High-sensitivity C-reactive protein, D-dimer, and homocysteine were excluded from the primary analysis because of substantial missingness. High Fazekas burden was used for descriptive analysis and was not entered together with the modified Fazekas total score to avoid redundant representation of the same imaging construct.

Four predictor sets were constructed. The clinical variable model was prespecified as the primary model. Three extended models were developed by adding the modified Fazekas score, inflammatory metabolic biomarkers, or both to the clinical variable model. The clinical variable model served as the reference for incremental value analysis. The integrated predictor set was used for secondary algorithm benchmarking and exploratory interpretation.

### 2.5 Data preprocessing and leakage control

Blank entries, null fields, not-a-number (NaN) values, and missing codes defined in the data dictionary were treated as missing values. Continuous variables were imputed using the median within each training fold, whereas categorical variables were imputed using the mode within each training fold. Categorical variables were transformed using one hot encoding. For binary variables, only one clinically meaningful coding form was retained to prevent complementary indicators from entering the model simultaneously. Standardisation was applied to linear models and K nearest neighbour models, but not to tree based models. High-sensitivity C-reactive protein, D-dimer, and homocysteine had substantial missingness, with missing rates of 45.1%, 49.2%, and 38.8%, respectively, and were excluded from the primary analysis.

All imputation, encoding, and standardisation procedures were embedded within the cross validation training process. When oversampling was used in sensitivity analyses, it was also performed only within the training folds. Collinearity among continuous variables was assessed using variance inflation factors and Spearman rank correlation analysis. Early rehabilitation need, discharge mRS score, discharge NIHSS score, discharge mRS score ≥2, discharge NIHSS score ≥3, rehabilitation treatment status, and follow up variables were excluded from the predictor sets to reduce the risk of information leakage.

### 2.6 Model development and internal validation

Eight regression and machine learning algorithms were evaluated, including logistic regression, least absolute shrinkage and selection operator logistic regression, elastic net logistic regression, classification and regression tree, random forest, kernel K nearest neighbour, naive Bayes, and light gradient boosting machine (LightGBM). Model development and internal validation were performed using stratified tenfold cross validation, with the random seed set to 2026. Within each outer training fold, hyperparameters were tuned using inner stratified fivefold cross validation, with the area under the receiver operating characteristic curve (AUROC) as the optimisation target. All candidate models used the same outer fold allocation to ensure consistent comparisons across algorithms.

The prespecified main analysis did not use the synthetic minority oversampling technique (SMOTE). SMOTE was used only in sensitivity analyses and was restricted to the training folds. No feature selection, imputation, standardisation, or oversampling was performed on the entire dataset before cross validation.

For the primary outcome, the prespecified primary analysis focused on the clinical variable model. Algorithm benchmarking was then performed using the integrated predictor set to evaluate model behaviour in the complete predictor space. AUROC was prespecified as the primary discrimination metric. When the difference in AUROC between candidate algorithms was less than 0.01, logistic regression or penalised logistic regression was preferred as the representative interpretable model to enhance clinical interpretability and translational potential. The primary clinical variable model was then compared with extended models that added imaging burden, inflammatory metabolic biomarkers, or both to assess incremental predictive value beyond clinical variables. Two simple logistic regression benchmarks, using NIHSS alone and NIHSS combined with inability to walk within 48 h, were evaluated using the same cross-validation framework as the primary model.

### 2.7 Model performance, calibration, and clinical utility

Model performance was evaluated using accuracy, F1 score, area under the precision–recall curve (AUPRC), sensitivity, specificity, positive predictive value, negative predictive value, and the Brier score. AUROC was prespecified as the primary discrimination metric. Because the outcome showed moderate class imbalance, AUPRC was also reported. All performance metrics were calculated from out of fold predicted probabilities generated in the outer cross validation loop. The 95% confidence intervals (CIs) for AUROC and AUPRC were estimated using bootstrap resampling.

Calibration was assessed using calibration curves, calibration intercept, calibration slope, and the Brier score. Logistic recalibration was performed when needed to explore potential improvement in calibration. Clinical utility was assessed using decision curve analysis, and net benefit was reported at prespecified threshold probabilities of 0.20, 0.30, and 0.40. Because tolerance for missed rehabilitation needs and excessive referral may differ across institutions, the potential clinical value of the model was judged by integrating discrimination, calibration, and decision curve findings.

### 2.8 Incremental value, sensitivity analyses, and model interpretation

Incremental value was assessed by comparing the primary clinical variable model with extended models that added the modified Fazekas score, inflammatory metabolic biomarkers, or both. Changes in AUROC, AUPRC, and Brier score were used to evaluate whether these additional predictor domains improved performance beyond clinical variables.

Sensitivity analyses focused on the primary outcome. These analyses included SMOTE-based modeling, complete-case analysis, exclusion of patients with pre-stroke mRS score of 2 or higher, and a non-functional predictor model excluding NIHSS score, mRS score at 24 h after admission, limb weakness, dysarthria, and inability to walk within 48 h. To establish a uniform prediction time point and evaluate potential incorporation bias, an admission fixed-landmark sensitivity analysis was performed using only variables available at admission. Functional measures recorded during the subsequent 24–48 h were excluded. Admission NIHSS alone and admission NIHSS combined with limb weakness were evaluated as benchmark models. A further reduced model excluded variables directly reflecting acute neurological deficits.

SHAP analysis was used to interpret model predictions. SHAP analysis was applied to the integrated extension model as an exploratory interpretation analysis, because this model included clinical variables, white matter hyperintensity burden, and inflammatory metabolic biomarkers. Global importance was ranked by mean absolute SHA*p* values, and individual-level explanations were shown using waterfall plots. SHA*p* values were interpreted as model-based contributions to prediction, not as causal effects.

All analyses were performed using Python 3.12. Data processing and statistical analyses were conducted using pandas 2.3 and NumPy 2.3. Regression and machine-learning models were developed and internally validated using scikit-learn 1.5 and LightGBM 4.6. SMOTE was implemented using imbalanced-learn 0.14.2, and model interpretation was performed using SHAP 0.52.

## 3 Results

### 3.1 Study population, baseline characteristics, and outcome distribution

**Figure 1:**
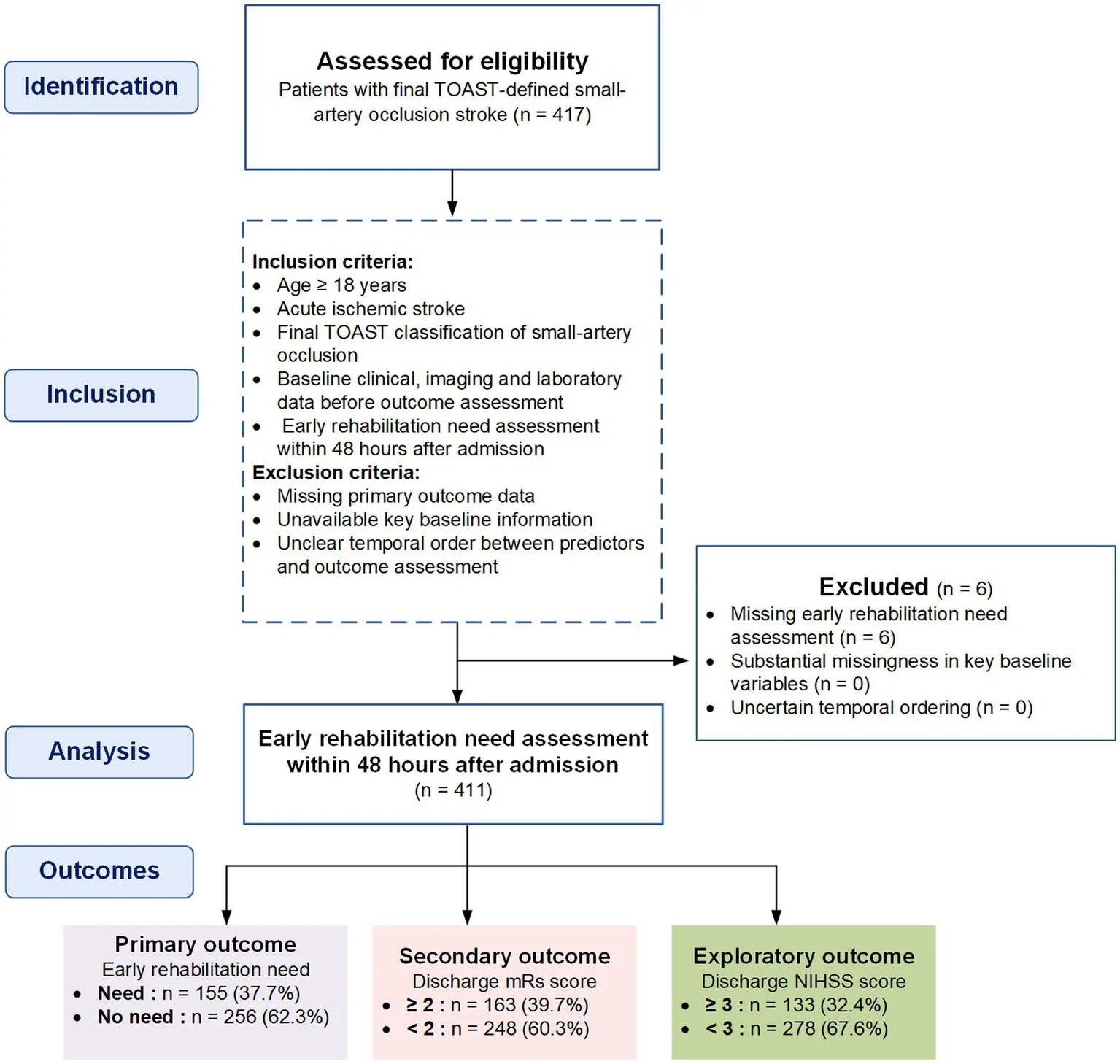
Study population and outcome definitions. Flowchart of patient selection and outcome definitions. Among 417 patients with final TOAST-defined small-artery occlusion stroke who met the prespecified clinical and imaging eligibility criteria, six were excluded because early rehabilitation need assessment was unavailable. No additional patients met the remaining prespecified exclusion criteria, leaving 411 patients in the final analysis. The primary outcome was early rehabilitation need during acute hospitalization, which occurred in 155 patients (37.7%). The secondary outcome was discharge functional dependence, defined as discharge mRS score ≥2, which occurred in 163 patients (39.7%). The exploratory outcome was residual neurological deficit at discharge, defined as discharge NIHSS score ≥3, which occurred in 133 patients (32.4%). TOAST, Trial of Org 10,172 in Acute Stroke Treatment; NIHSS, National Institutes of Health Stroke Scale; mRS, Modified Rankin Scale.

A total of 417 patients with small artery occlusion acute ischemic stroke were screened. Six were excluded because early rehabilitation need assessment was unavailable, leaving 411 patients in the final analysis. The patient selection process is shown in Figure 1.

Baseline characteristics stratified by early rehabilitation need are shown in Table 1. Patients with early rehabilitation need had greater neurological and functional impairment, as reflected by higher initial NIHSS scores, higher mRS scores at 24 h after admission, more frequent limb weakness, and a higher proportion of inability to walk within 48 h. Admission to a stroke unit and university level education or higher were also more common in this group. In contrast, vascular risk factors, vital signs, white matter hyperintensity burden, and inflammatory metabolic biomarkers were broadly comparable between groups. Similar patterns of greater neurological and functional impairment were observed in patients with discharge mRS score ≥2, with additional differences in neutrophil count and neutrophil to lymphocyte ratio, as shown in Supplementary Table S1.

**Table 1:** Baseline characteristics of the study population stratified by early rehabilitation need.

Variable	No early rehabilitation need (*n* = 256)	Early rehabilitation need (*n* = 155)	*p* value
Demographic characteristics			
Age, years	56.08 ± 11.61	58.54 ± 13.02	0.054
Male sex	184 (71.9)	109 (70.3)	0.736
University education or higher	51 (19.9)	48 (31.0)	0.011
Medical insurance			0.416
New rural cooperative medical insurance	1 (0.4)	1 (0.6)	
Self-pay	60 (23.4)	28 (18.1)	
Urban employee/resident or public insurance	195 (76.2)	126 (81.3)	
Vascular risk factors			
Hypertension	154 (60.2)	105 (67.7)	0.123
Diabetes mellitus	42 (16.4)	33 (21.3)	0.214
Prior ischemic stroke	29 (11.3)	26 (16.8)	0.116
Drinking status			0.417
Current drinker	53 (20.7)	24 (15.5)	
Former drinker	2 (0.8)	3 (1.9)	
Never drinker	186 (72.7)	120 (77.4)	
Unknown	15 (5.9)	8 (5.2)	
Stroke severity and early function			
Initial NIHSS score	3.00 [1.00, 4.00]	4.00 [2.00, 6.00]	<0.001
mRS score at 24 h after admission	2.00 [1.00, 3.00]	3.00 [2.00, 3.00]	<0.001
Initial GCS score	15.00 [15.00, 15.00]	15.00 [15.00, 15.00]	0.410
Limb weakness	157 (61.3)	122 (78.7)	<0.001
Dysarthria	80 (31.2)	55 (35.5)	0.376
Unable to walk within 48 h	12 (4.7)	34 (21.9)	<0.001
Admission to stroke unit	134 (52.3)	109 (70.3)	<0.001
Initial systolic blood pressure, mmHg	157.36 ± 27.41	155.70 ± 26.82	0.545
Initial diastolic blood pressure, mmHg	92.00 [80.00, 102.00]	89.00 [78.50, 100.50]	0.202
Initial pulse rate, beats/min	83.17 ± 13.44	80.74 ± 13.51	0.078
Initial body temperature, °C	36.50 [36.27, 36.60]	36.50 [36.40, 36.55]	0.508
Onset-to-arrival time, h	18.92 [5.27, 55.70]	16.76 [5.72, 40.67]	0.316
White matter hyperintensity burden			
Modified Fazekas score	2.00 [1.00, 3.00]	2.00 [1.00, 3.00]	0.565
High Fazekas burden	162 (63.3)	100 (64.5)	0.801
Inflammatory metabolic biomarkers			
White blood cell count	7.18 [5.96, 8.70]	7.10 [5.92, 8.34]	0.805
Neutrophil count	4.58 [3.61, 5.68]	4.38 [3.73, 5.78]	0.961
Lymphocyte count	1.87 [1.40, 2.27]	1.83 [1.43, 2.31]	0.794
Neutrophil-to-lymphocyte ratio	2.47 [1.81, 3.53]	2.58 [1.80, 3.58]	0.827
Fibrinogen	2.90 [2.52, 3.30]	2.96 [2.66, 3.39]	0.199
Fasting glucose	5.20 [4.63, 6.03]	5.24 [4.69, 6.34]	0.741
Glycated haemoglobin	5.80 [5.50, 6.60]	6.00 [5.50, 6.85]	0.188
LDL cholesterol	3.06 ± 0.86	3.14 ± 0.89	0.366
Serum uric acid	376.07 ± 108.44	364.98 ± 92.21	0.273
Serum creatinine	74.70 [60.85, 88.83]	76.00 [63.00, 89.55]	0.516
Blood urea nitrogen	5.14 [4.19, 5.95]	5.09 [4.17, 5.98]	0.843
Alanine aminotransferase	20.00 [15.00, 31.00]	20.00 [14.00, 27.00]	0.305

Values are presented as mean ± standard deviation, median [interquartile range], or n (%), as appropriate. P values were calculated using Student’s *t* test, Mann Whitney U test, chi-square test, or Fisher’s exact test, as appropriate. NIHSS, National Institutes of Health Stroke Scale; mRS, modified Rankin Scale; GCS, Glasgow Coma Scale; LDL, low density lipoprotein.

Outcome distribution and representative models are summarised in Table 2. Early rehabilitation need occurred in 155 patients, accounting for 37.7% of the cohort. Discharge mRS score ≥2 occurred in 163 patients, and discharge NIHSS score ≥3 occurred in 133 patients. The three outcomes were modelled and internally validated separately.

**Table 2:** Outcome distribution and representative algorithm performance in the integrated benchmarking analysis.

Outcome	Role	Positive, *n*/*N* (%)	Model	AUROC	95% CI	AUPRC	Brier
Early rehabilitation need	Primary	155/411 (37.7%)	Logistic regression	0.670	0.614 to 0.719	0.544	0.216
Discharge mRS ≥ 2	Secondary	163/411 (39.7%)	Random forest	0.788	0.743 to 0.830	0.683	0.205
Discharge NIHSS ≥3	Exploratory	133/411 (32.4%)	Random forest	0.818	0.774 to 0.859	0.683	0.182

The integrated predictor set included clinical variables, modified Fazekas score, and inflammatory metabolic biomarkers. AUROC was used as the primary selection metric. For the primary outcome, logistic regression was selected as a representative interpretable model because its AUROC was within 0.01 of the best performing model. For secondary and exploratory outcomes, the model with the highest AUROC was selected. CI, confidence interval; AUROC, area under the receiver operating characteristic curve; AUPRC, area under the precision recall curve; NIHSS, National Institutes of Health Stroke Scale; mRS, modified Rankin Scale.

### 3.2 Correlation structure of continuous predictors

**Figure 2:**
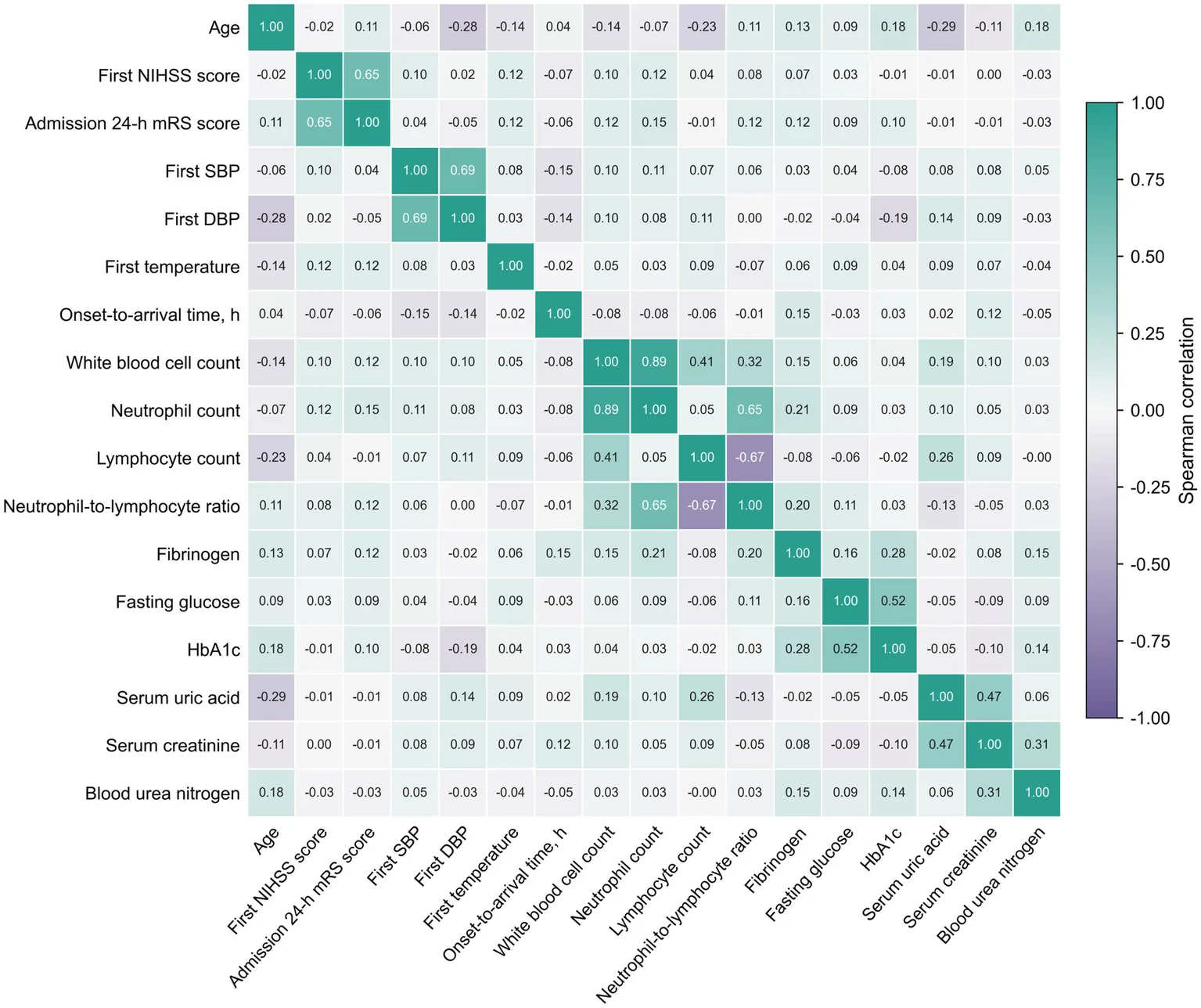
Correlation structure of retained continuous predictors. Heatmap showing pairwise Spearman correlations among retained continuous predictors after preprocessing. Colour intensity indicates the direction and magnitude of each correlation, and numbers within cells indicate correlation coefficients. The strongest positive correlation was observed between white blood cell count and neutrophil count. Moderate positive correlations were observed between initial systolic and diastolic blood pressure, and between initial NIHSS score and mRS score at 24 h after admission. Other correlations were generally weak, with no evidence of widespread severe collinearity among retained continuous predictors. NIHSS, National Institutes of Health Stroke Scale; mRS, modified Rankin Scale; HbA1c, glycated haemoglobin A1c.

The correlation structure among retained continuous predictors is shown in Figure 2. The strongest positive correlation was observed between white blood cell count and neutrophil count. Moderate positive correlations were observed between initial systolic and diastolic blood pressure, and between initial NIHSS score and mRS score at 24 h after admission. Correlations among the remaining continuous variables were generally weak, with no evidence of widespread severe collinearity.

### 3.3 Secondary algorithm benchmarking and discrimination in the integrated predictor set

**Figure 3:**
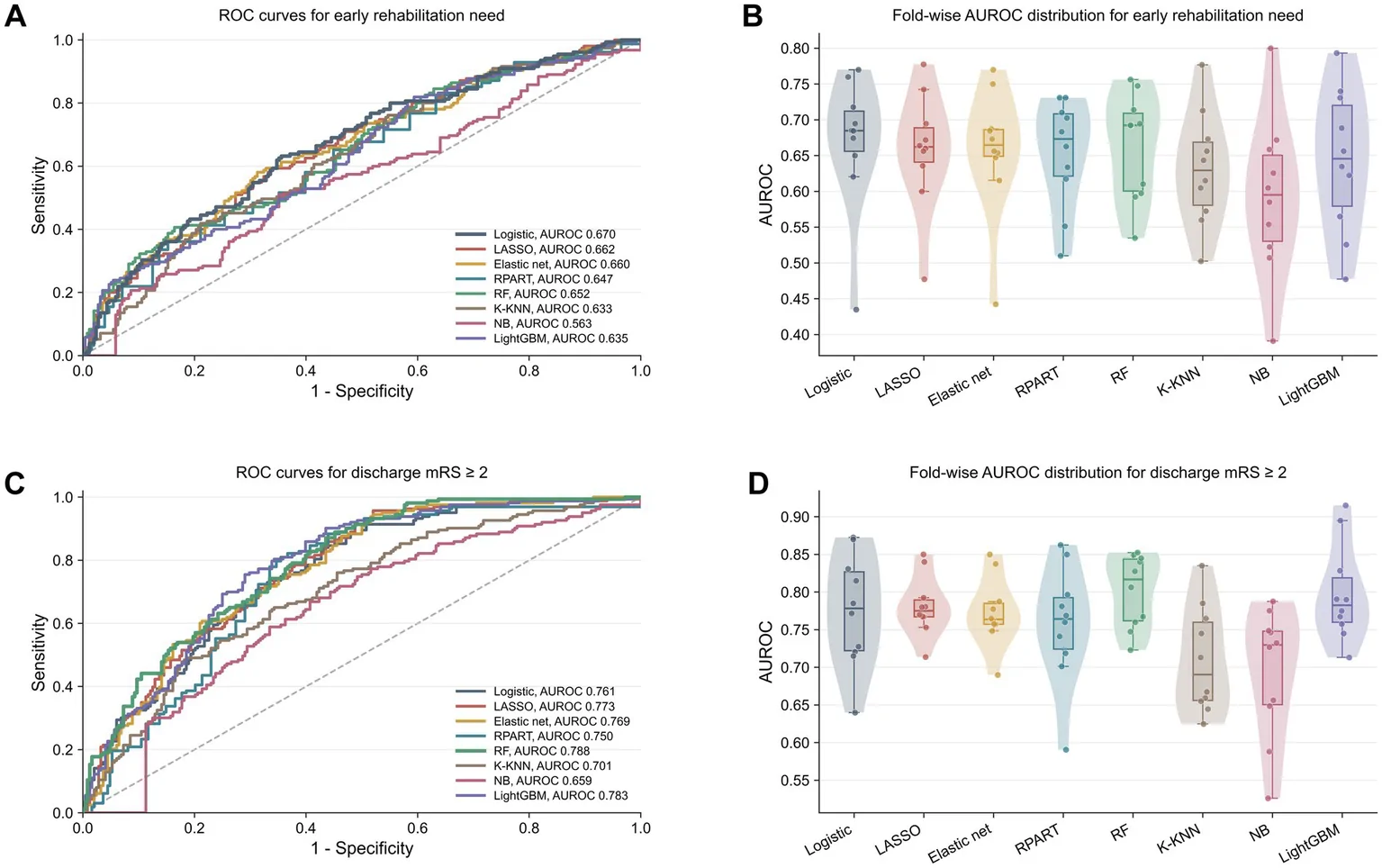
ROC based benchmarking of candidate algorithms in the integrated predictor set. **(A)** Receiver operating characteristic curves of candidate algorithms for predicting early rehabilitation need in the integrated predictor set. **(B)** Fold wise AUROC distributions of candidate algorithms for early rehabilitation need under stratified tenfold cross validation. **(C)** Receiver operating characteristic curves of candidate algorithms for predicting discharge mRS score ≥2. **(D)** Fold wise AUROC distributions of candidate algorithms for discharge mRS score ≥2. The diagonal dashed line in each ROC plot indicates the reference line. For the primary outcome, logistic regression was selected as the representative interpretable model because its discrimination was comparable to that of the best performing algorithm. For the secondary outcome, random forest achieved the highest AUROC and was selected as the representative model. ROC, receiver operating characteristic; AUROC, area under the receiver operating characteristic curve; mRS, modified Rankin Scale.

**Figure 4:**
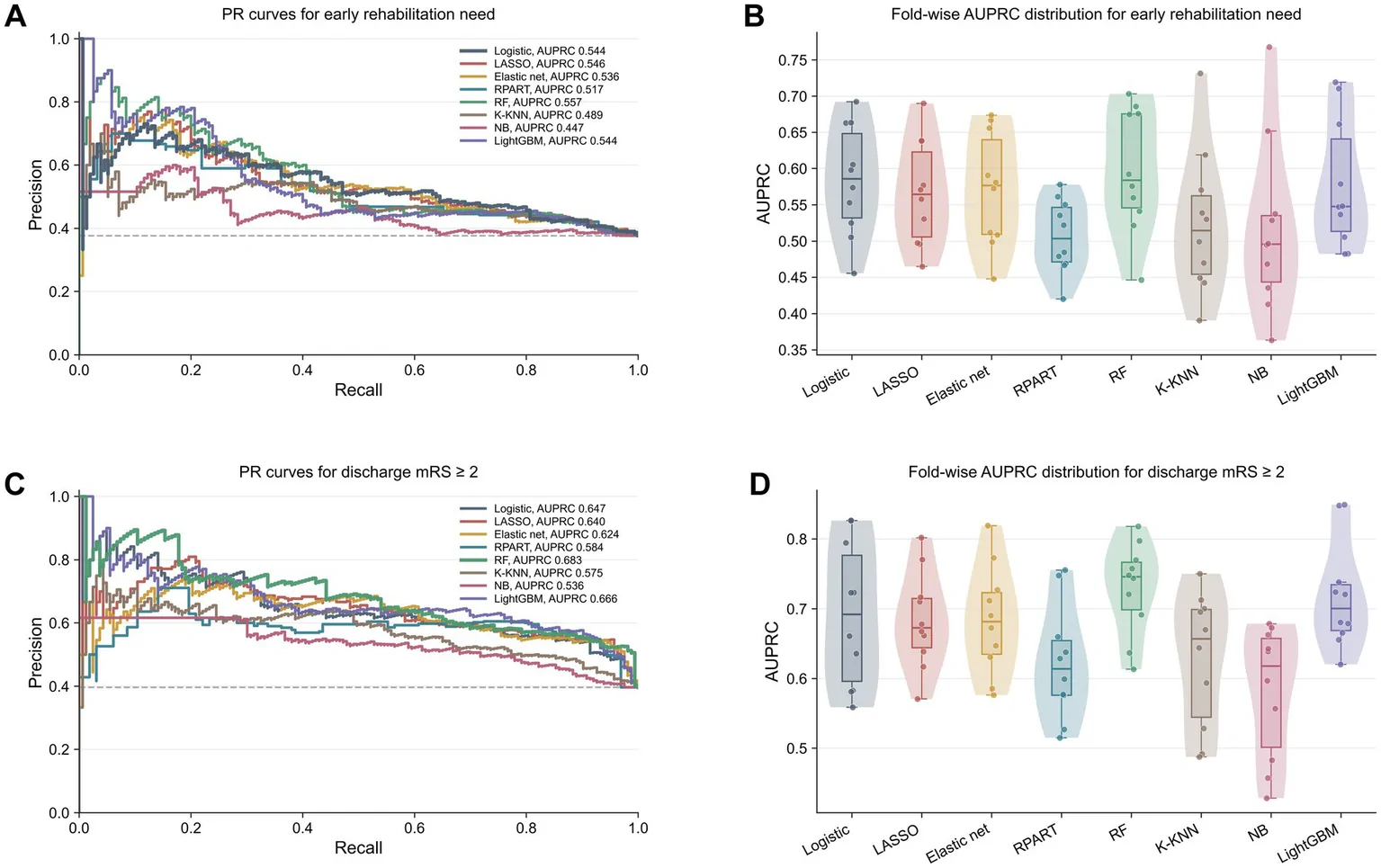
Precision recall benchmarking of candidate algorithms in the integrated predictor set. **(A)** Precision recall curves of candidate algorithms for predicting early rehabilitation need in the integrated predictor set. **(B)** Fold-wise AUPRC distributions of candidate algorithms for early rehabilitation need under stratified tenfold cross validation. **(C)** Precision recall curves of candidate algorithms for predicting discharge mRS score ≥2. **(D)** Fold-wise AUPRC distributions of candidate algorithms for discharge mRS score ≥2. The horizontal dashed line in each precision recall plot indicates the prevalence of the corresponding outcome. AUPRC values are shown in the figure legends. Precision recall curves were generated using out-of-fold predicted probabilities without artificial endpoint extension. AUPRC, area under the precision recall curve; mRS, modified Rankin Scale.

Model performance for the primary and secondary outcomes is summarised in Tables 2, 3. For the primary outcome, logistic regression was selected as the representative model in the integrated predictor set because it showed stable discrimination and good interpretability. This model yielded an AUROC of 0.670, a 95% CI of 0.614 to 0.719, an AUPRC of 0.544, and a Brier score of 0.216. Receiver operating characteristic (ROC) curves and fold-wise AUROC distributions are shown in Figures 3A,B. Precision-recall curves and fold-wise AUPRC distributions are shown in Figures 4A,B. The corresponding precision-recall curves and fold-wise AUPRC distributions for discharge mRS score ≥2 are shown in Figures 4C,D.

**Table 3:** Cross-validated classification performance of representative models.

Outcome	Model	Accuracy	F1 score	Sensitivity	Specificity	Precision	NPV
Early rehabilitation need	Logistic regression	0.669	0.447	0.355	0.859	0.604	0.688
Discharge mRS ≥ 2	Random forest	0.655	0.311	0.196	0.956	0.744	0.644

Performance was estimated using stratified 10-fold cross validation and out of fold predictions. The primary analysis did not use SMOTE. AUROC, AUPRC, and Brier score are presented in Table 2 to avoid duplication. Complete candidate-model performance, including the exploratory discharge NIHSS ≥3 outcome, is provided in the Supplementary materials. NPV, negative predictive value; NIHSS, National Institutes of Health Stroke Scale; mRS, modified Rankin Scale; SMOTE, synthetic minority oversampling technique.

At the default classification threshold, the representative early rehabilitation need model had an accuracy of 0.669, an F1 score of 0.447, a sensitivity of 0.355, a specificity of 0.859, a positive predictive value of 0.604, and a negative predictive value of 0.688.

For discharge mRS score ≥2, the random forest model yielded an AUROC of 0.788, a 95% CI of 0.743 to 0.830, an AUPRC of 0.683, and a Brier score of 0.205. For discharge NIHSS score ≥3, the random forest model yielded an AUROC of 0.818, a 95% CI of 0.774 to 0.859, an AUPRC of 0.683, and a Brier score of 0.182. Complete candidate model results are provided in Supplementary Table S2, and the corresponding ROC and precision-recall curves are shown in Supplementary Figure S1.

### 3.4 Calibration and decision curve analysis

**Figure 5:**
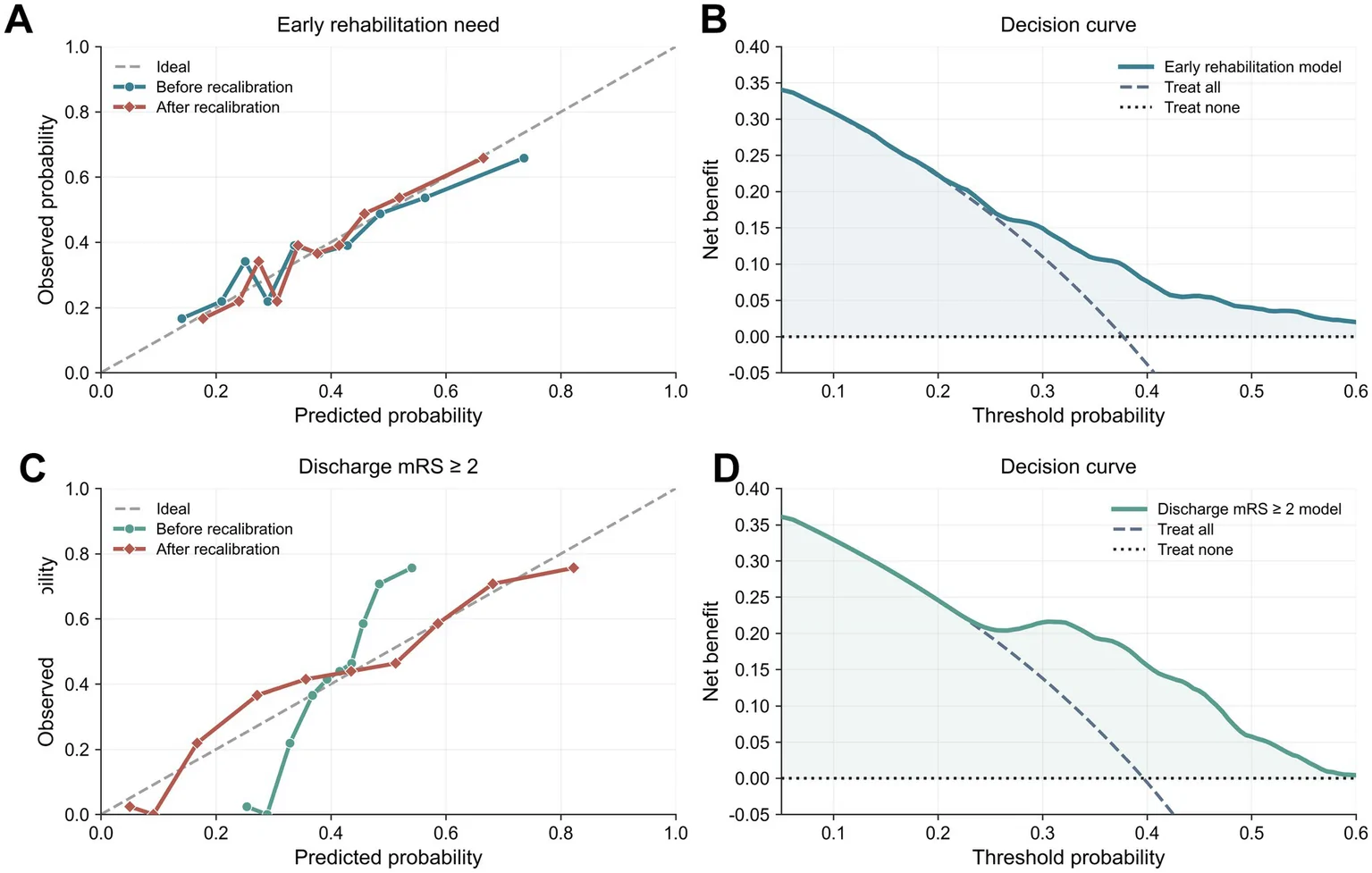
Calibration and clinical utility of representative models in the integrated predictor set. **(A)** Calibration curve for the representative logistic regression model for early rehabilitation need before and after logistic recalibration. **(B)** Decision curve analysis for the representative early rehabilitation need model across clinically relevant threshold probabilities. **(C)** Calibration curve for the representative random forest model for discharge mRS score ≥ 2 before and after logistic recalibration. **(D)** Decision curve analysis for the representative discharge mRS score ≥ 2 model. The diagonal dashed line in each calibration plot indicates perfect calibration. In decision curve analysis, the solid line represents the model, the dashed line represents the treat all strategy, and the dotted line represents the treat none strategy. Shaded areas indicate positive net benefit across threshold probabilities. mRS, Modified Rankin Scale.

Calibration curves and decision curve analyses for the primary and secondary outcome models are shown in Figure 5 and Table 4. For early rehabilitation need, the logistic regression model had a Brier score of 0.216, a calibration intercept of −0.122, and a calibration slope of 0.776. Decision curve analysis showed net benefits of 0.224, 0.148, and 0.076 at threshold probabilities of 0.20, 0.30, and 0.40, respectively.

**Table 4:** Calibration and clinical utility of representative models for the primary and secondary outcomes.

Outcome	Model	AUROC (95% CI)	AUPRC	Brier	Intercept	Slope	NB 0.20	NB 0.30	NB 0.40
Early rehabilitation need	Logistic regression	0.670 (0.614 to 0.719)	0.544	0.216	−0.122	0.776	0.224	0.148	0.076
Discharge mRS ≥ 2	Random forest	0.788 (0.743 to 0.830)	0.683	0.205	0.223	0.538	0.246	0.216	0.156

Calibration intercept and slope were estimated from out of fold predicted probabilities before recalibration. Net benefit was calculated using decision curve analysis at prespecified threshold probabilities. Internal validation was based on stratified 10-fold cross validation. CI, confidence interval; AUROC, area under the receiver operating characteristic curve; AUPRC, area under the precision recall curve; mRS, modified Rankin Scale; NB, net benefit.

For discharge mRS score ≥2, the random forest model had a Brier score of 0.205, a calibration intercept of 0.223, and a calibration slope of 0.538. At threshold probabilities of 0.20, 0.30, and 0.40, the corresponding net benefits were 0.246, 0.216, and 0.156, respectively.

### 3.5 Incremental predictive value and sensitivity analyses

**Figure 6:**
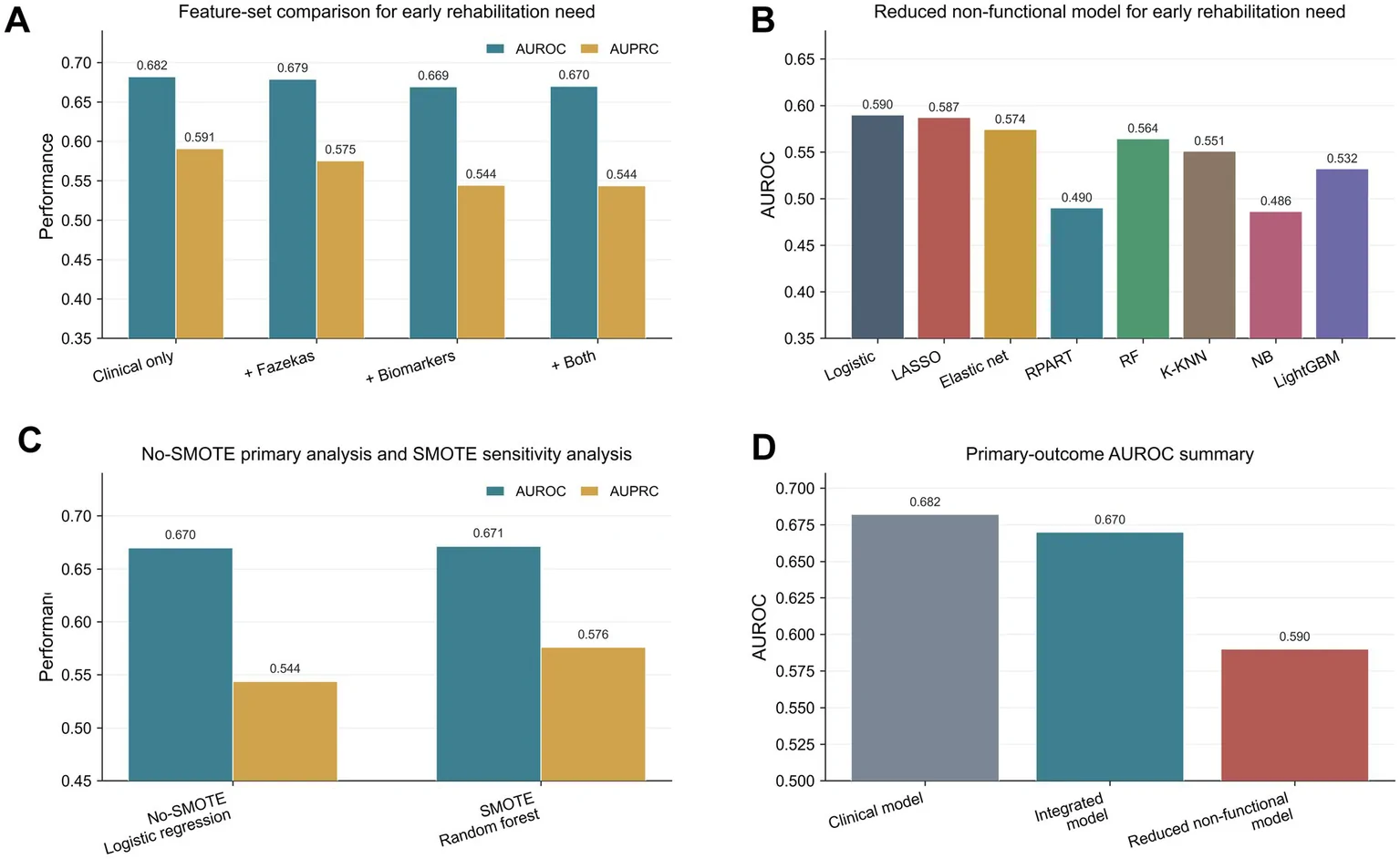
Predictor set comparison and sensitivity analyses for early rehabilitation need. **(A)** Predictor set comparison using nested logistic regression models for early rehabilitation need. The primary clinical variable model was compared with extended models incorporating the modified Fazekas score, inflammatory metabolic biomarkers, or both. **(B)** AUROC performance of candidate algorithms in the non-functional predictor model after exclusion of direct neurological severity and early functional status predictors. **(C)** Comparison between the prespecified no SMOTE analysis and the SMOTE-based sensitivity analysis. **(D)** AUROC summary comparing the primary clinical variable model, the integrated extension model, and the non-functional predictor model for early rehabilitation need. Adding the modified Fazekas score and inflammatory metabolic biomarkers did not improve discrimination beyond clinical variables. The non-functional predictor model showed lower discrimination, suggesting that early rehabilitation need prediction was mainly driven by neurological severity and early functional impairment. AUROC, area under the receiver operating characteristic curve; AUPRC, area under the precision recall curve; SMOTE, synthetic minority oversampling technique.

Results of the incremental value analysis and sensitivity analyses for early rehabilitation need are shown in Figure 6 and Supplementary Tables S3, S4. In the prespecified predictor-set comparison, the primary clinical variable model yielded an AUROC of 0.682 and an AUPRC of 0.591. After the modified Fazekas score was added, the AUROC and AUPRC were 0.679 and 0.575, respectively. After inflammatory-metabolic biomarkers were added, the AUROC and AUPRC were 0.669 and 0.544, respectively. When both predictor domains were added, the integrated extension model yielded an AUROC of 0.670 and an AUPRC of 0.544. The NIHSS alone and NIHSS plus early walking ability benchmark models yielded AUROCs of 0.662 and 0.681, respectively, compared with 0.682 for the primary clinical-variable model.

Sensitivity analyses supported the robustness of the main findings. In the main analysis without SMOTE, the logistic regression model yielded an AUROC of 0.670, an AUPRC of 0.544, and a Brier score of 0.216. In the SMOTE-based sensitivity analysis, the random forest model yielded an AUROC of 0.671, an AUPRC of 0.576, and a Brier score of 0.226. Complete-case analysis yielded an AUROC of 0.650 and an AUPRC of 0.577. After excluding patients with pre-stroke mRS score ≥2, the logistic regression model yielded an AUROC of 0.670, an AUPRC of 0.574, and a Brier score of 0.214. In the non-functional predictor model, which excluded NIHSS score, mRS score at 24 h after admission, limb weakness, dysarthria, and inability to walk within 48 h, the logistic regression model showed lower performance, with an AUROC of 0.590, an AUPRC of 0.441, and a Brier score of 0.238.

In the admission fixed-landmark sensitivity analysis, the clinical-variable model yielded an AUROC of 0.623 (95% CI, 0.569–0.654), an AUPRC of 0.481 (95% CI, 0.411–0.568), and a Brier score of 0.231. Admission NIHSS alone yielded an AUROC of 0.612 and an AUPRC of 0.527, whereas NIHSS combined with limb weakness yielded an AUROC of 0.623 and an AUPRC of 0.524. After variables directly reflecting acute neurological deficits were excluded, the AUROC decreased to 0.507 and the AUPRC was 0.406.

### 3.6 SHAP based model interpretation

**Figure 7:**
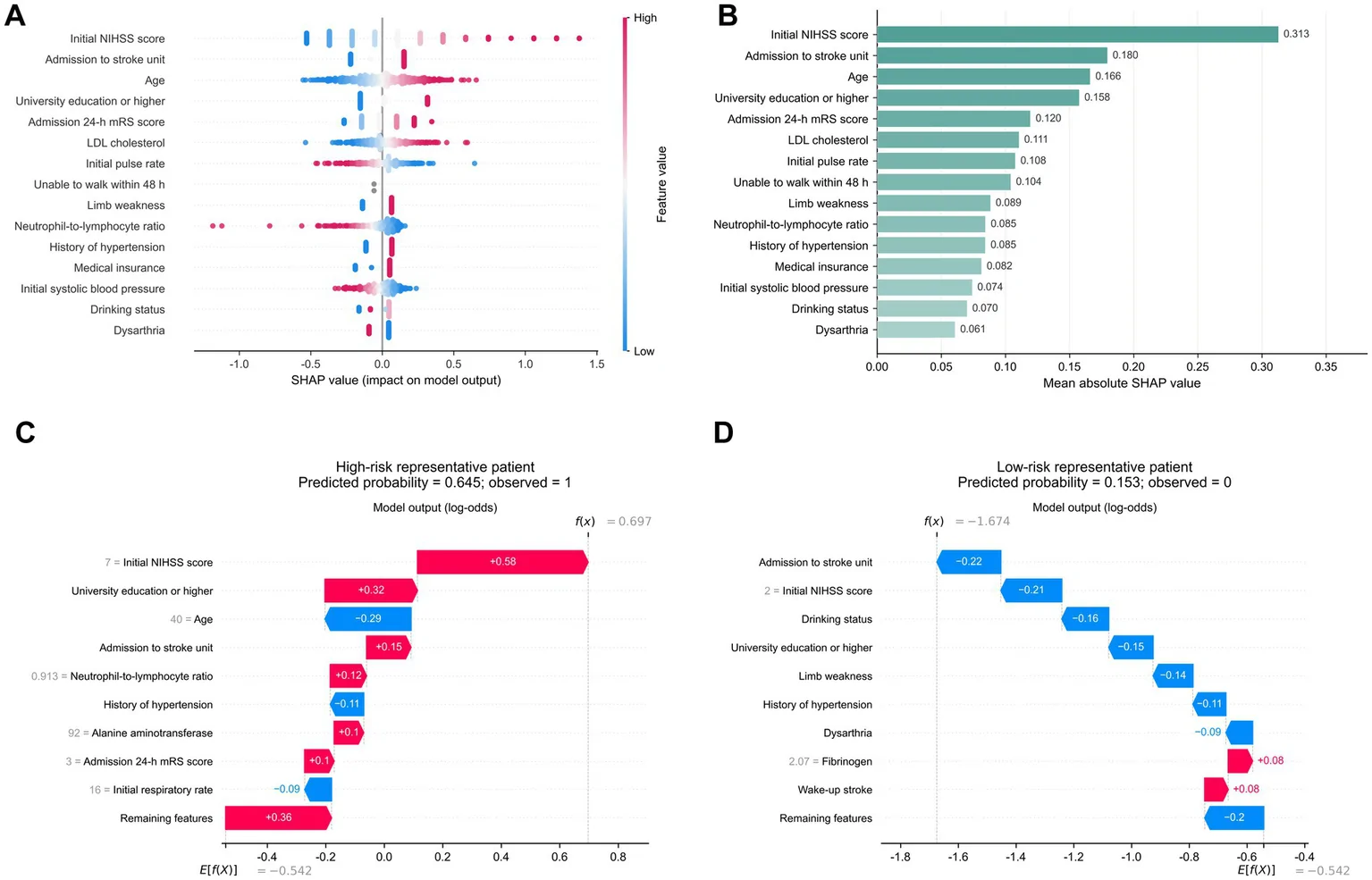
SHAP based interpretation of the integrated extension model for early rehabilitation need. **(A)** SHAP summary plot showing the distribution of variable contributions in the integrated extension model for early rehabilitation need. Each dot represents one patient, and colour denotes variable values from low to high. **(B)** Mean absolute SHAP value ranking of the leading predictors. Initial NIHSS score contributed most to model output, followed by admission to a stroke unit, age, university level education or higher, mRS score at 24 h after admission, LDL cholesterol, initial pulse rate, inability to walk within 48 h, limb weakness, neutrophil to lymphocyte ratio, history of hypertension, medical insurance, initial systolic blood pressure, drinking status, and dysarthria. **(C)** SHAP waterfall plot for a representative patient with higher predicted risk. **(D)** SHAP waterfall plot for a representative patient with lower predicted risk. SHAP values indicate model based variable contributions and should not be interpreted as causal effects. SHAP, Shapley additive explanations; NIHSS, National Institutes of Health Stroke Scale; mRS, modified Rankin Scale; LDL, low density lipoprotein.

SHAP based interpretation of the integrated extension model is shown in Figure 7. Initial NIHSS score had the largest contribution to model output, followed by admission to a stroke unit, age, university level education or higher, mRS score at 24 h after admission, low density lipoprotein cholesterol, initial pulse rate, inability to walk within 48 h, limb weakness, and neutrophil to lymphocyte ratio.

The leading predictors mainly reflected stroke severity, early functional status, care pathway or admission related factors, demographic and sociodemographic characteristics, metabolic indicators, physiological measures, and inflammatory markers. Individual SHAP waterfall plots showed variable contributions in representative patients with higher and lower predicted risk. SHAP values indicate the direction and magnitude of variable contributions to model output and should not be interpreted as causal effects.

## 4 Discussion

This study developed and internally validated a clinical variable model for identifying early rehabilitation need during acute hospitalization in patients with small artery occlusion stroke. We also assessed whether white matter hyperintensity burden and inflammatory metabolic biomarkers added predictive value beyond routine clinical variables. Early rehabilitation need was common, affecting more than one-third of patients. The clinical variable model showed the best overall performance, while models incorporating the modified Fazekas score, inflammatory metabolic biomarkers, or both provided no stable improvement. SHAP analysis of the integrated extension model indicated that the leading predictors mainly reflected neurological severity, early functional status, and care pathway information. These findings suggest that routinely available clinical variables carried the dominant predictive signal for early rehabilitation need in this population.

Small artery occlusion stroke is often viewed as a mild subtype because lesions are usually small, initial neurological deficits are limited, and short-term outcomes are generally favourable (17). However, in our cohort, more than one-third of patients were judged to require further structured rehabilitation assessment during acute hospitalization. This finding indicates that clinically meaningful functional limitation can occur even when this stroke subtype appears mild. Perforator-territory infarcts may affect strategic motor pathways in the internal capsule, pons, basal ganglia, and corona radiata (18). Such involvement can lead to limb weakness, gait disturbance, and reduced independence in activities of daily living (19). Stroke subtype and lesion size alone are therefore insufficient for estimating early rehabilitation need. Bedside neurological assessment and early functional evaluation should remain central to rehabilitation triage.

The model showed moderate discriminatory ability, rather than high accuracy, for identifying early rehabilitation need. In the integrated extension analysis, the representative logistic regression model achieved an AUROC of 0.670, an AUPRC of 0.544, and a Brier score of 0.216. At the default classification threshold, the model showed relatively low sensitivity but high specificity. This threshold-dependent pattern suggests that the model may be better suited to ruling out lower-risk patients than to capturing all patients with early rehabilitation need (20). Although calibration was not ideal, decision curve analysis showed positive net benefit across the prespecified range of threshold probabilities (21). Because tolerance for missed rehabilitation needs and unnecessary referrals may vary across institutions, risk thresholds should be locally calibrated according to rehabilitation resources, referral capacity, and clinical priorities before implementation (22).

Routine clinical variables accounted for most of the predictive information for early rehabilitation need (23). SHAP analysis of the integrated extension model indicated that the leading predictors clustered around neurological severity, early functional status, and care pathway characteristics. Stroke unit admission and educational level may reflect differences in care pathways, access to healthcare, and sociodemographic characteristics rather than direct markers of stroke severity. These variables should therefore be interpreted as contextual factors associated with rehabilitation triage rather than disease-specific predictors. NIHSS score, 24-h mRS score, walking ability, and limb weakness are clinically plausible predictors because they directly capture acute neurological impairment and mobility restriction (24, 25). In small artery occlusion stroke, involvement of strategic motor pathways may compromise walking, transfer, and activities of daily living, even when the NIHSS score is relatively low (26). The decline in AUROC to 0.590 in the non-functional predictor model further supports the interpretation that early neurological severity and functional status provided the main predictive signal. The admission fixed-landmark model showed lower discrimination than the primary early in-hospital triage model. After variables directly reflecting acute neurological deficits were excluded, the AUROC decreased to 0.507. These findings indicate that most of the predictive signal was derived from acute neurological status. They also suggest that some early functional predictors were conceptually close to the clinician-assigned rehabilitation need and may have increased the apparent performance of the primary model. The multivariable clinical model showed slightly higher discrimination than NIHSS alone but performed similarly to the model combining NIHSS with early walking ability. This finding suggests that most predictive information was captured by routine bedside neurological and mobility assessments.

The modified Fazekas score and inflammatory-metabolic biomarkers provided no consistent incremental predictive value beyond clinical variables. For early rehabilitation need, the clinical variable model achieved an AUROC of 0.682, whereas extended models incorporating the modified Fazekas score, inflammatory-metabolic biomarkers, or both yielded AUROC values of 0.669 to 0.679. This pattern may reflect the clinically defined nature of the outcome (27). Early rehabilitation need primarily reflects acute limitations in motor function, walking ability, swallowing, and activities of daily living, all of which are directly assessed at the bedside (28). In contrast, white matter hyperintensity burden represents a chronic imaging marker of cerebral small vessel disease (29). Inflammatory-metabolic biomarkers may reflect systemic inflammation, vascular risk burden, metabolic status, or post-stroke immune responses (30). Therefore, these variables may provide limited additional information beyond direct bedside neurological and functional assessment during acute hospitalization.

Models for discharge mRS score ≥2 and discharge NIHSS score ≥3 yielded higher AUROC values than the model for early rehabilitation need. This finding suggests that the same early predictor set contained prognostic information for discharge functional dependence and residual neurological deficits (31). These outcomes are closer to traditional stroke prognostic endpoints and may reflect neurological recovery, in-hospital complications, treatment course, and rehabilitation exposure (32). Therefore, they were analysed as secondary and exploratory endpoints to examine model performance across different rehabilitation-related outcomes, rather than as the primary target of this study.

In practice, the decision threshold should reflect the relative consequences of missed rehabilitation needs and unnecessary referrals. A lower threshold may be preferred when minimizing missed needs is the priority, whereas a higher threshold may be appropriate when rehabilitation assessment capacity is limited. Therefore, the threshold should be selected within a clinically plausible range based on local resources, referral capacity, and threshold-specific net benefit, and should be locally calibrated before implementation (33). Although the specific referral pathway may vary across healthcare settings, the core assessment domains neurological deficits, motor function, activities of daily living, gait ability, and swallowing function are routinely evaluated in stroke care and are likely to be generalizable (34). The use of a structured assessment within 48 h, standardized assessor training, and adjudication of disagreements may also be transferable. However, decision thresholds, staffing arrangements, and referral pathways should be adapted to local rehabilitation resources (35).

This study has several implications for early rehabilitation triage. By focusing on small artery occlusion stroke, a subtype often perceived as clinically mild, we showed that early rehabilitation need was frequent during acute hospitalization. Incremental analysis further suggested that routinely available clinical variables carried most of the predictive signal, whereas imaging burden and inflammatory-metabolic biomarkers provided no consistent incremental value. SHAP analysis enhanced model transparency by ranking the variables with the greatest contributions to model output. Together, these findings support the future development and external validation of a simplified bedside clinical model for early rehabilitation triage in patients with small artery occlusion stroke.

## 5 Limitations

This study has several limitations. First, as a single-centre retrospective study with a relatively limited sample size, the model underwent only internal validation. The results may have been influenced by the local patient population, rehabilitation assessment procedures, and medical resource allocation. Future multicentre external validation is required to assess model stability across different hospitals and rehabilitation pathways. Second, this study lacked systematic quantification of the overall cerebral small vessel disease burden. Lacunes, cerebral microbleeds, and enlarged perivascular spaces were not fully incorporated. Consequently, the modified Fazekas score was used as the only imaging marker of white matter hyperintensity burden, which may not fully capture the overall imaging burden of cerebral small vessel disease. Third, because high-resolution vessel-wall imaging was not systematically available, occult non-stenotic parent-artery plaque or branch atheromatous disease could not be reliably identified in all patients. This may have introduced some uncertainty into the etiological classification of small-artery occlusion. High-sensitivity C-reactive protein, D-dimer, and homocysteine had substantial missingness and were excluded from the primary analysis, which may have limited the evaluation of their incremental predictive value.

## 6 Conclusion

This study showed that early rehabilitation need was common during acute hospitalization among patients with small artery occlusion stroke. Routinely available clinical variables provided the main predictive signal for early rehabilitation need, whereas white matter hyperintensity burden and inflammatory metabolic biomarkers added no stable incremental value. The admission fixed-landmark sensitivity analysis showed lower discrimination, with the AUROC decreasing from 0.623 to 0.507 after variables directly reflecting acute neurological deficits were excluded. These findings suggest that most of the predictive information was derived from acute neurological status and reinforce the need for cautious interpretation of the primary model. The primary clinical variable model may inform early in-hospital rehabilitation triage as an adjunct to clinical assessment, but multicentre external validation and prospective impact assessment are required before broader clinical implementation.

## Data Availability

The raw data supporting the conclusions of this article will be made available by the authors, without undue reservation.
