# Supplementary material for: Clinical variables provide the main predictive signal for early in-hospital rehabilitation triage after small-artery occlusion stroke: development and internal validation of an interpretable model
Source: Front Neurol. 2026 Sep 11;17:1910602. doi: 10.3389/fneur.2026.1910602 (PMC13612356; doi:10.3389/fneur.2026.1910602)
Supplement: Supplementary file 1 [file Supplementary_file_1.docx]

Supplementary Material

# 1 Supplementary Figures and Tables

## 1.1 Supplementary Figures

**Supplementary Fig. S1. Exploratory outcome performance for discharge NIHSS score ≥3**

a, Receiver operating characteristic curves of candidate models for predicting discharge NIHSS score ≥3. b, Fold wise AUROC distributions of candidate models for discharge NIHSS score ≥3 under stratified 10-fold cross validation. c, Precision recall curves of candidate models for predicting discharge NIHSS score ≥3. d, Fold wise AUPRC distributions of candidate models for discharge NIHSS score ≥3. The exploratory outcome was residual neurological deficit at discharge, defined as discharge NIHSS score ≥3. The horizontal dashed line in the precision recall plot indicates the prevalence of discharge NIHSS score ≥3. AUROC, area under the receiver operating characteristic curve; AUPRC, area under the precision recall curve; NIHSS, National Institutes of Health Stroke Scale.

## 1.2 Supplementary Tables

**Table S1. Baseline characteristics according to discharge mRS score ≥ 2**

| **Variable** | **Discharge mRS < 2 (n = 248)** | **Discharge mRS ≥ 2 (n = 163)** | **P value** |
| --- | --- | --- | --- |
| ***Demographic characteristics*** | | | |
| Age, years | 55.93 ± 11.14 | 58.64 ± 13.54 | 0.035 |
| Male sex | 173 (69.8) | 120 (73.6) | 0.397 |
| University education or higher | 56 (22.6) | 43 (26.4) | 0.378 |
| Medical insurance |  |  | 0.919 |
| New rural cooperative medical insurance | 1 (0.4) | 1 (0.6) |  |
| Self-pay | 52 (21.0) | 36 (22.1) |  |
| Urban employee/resident or public insurance | 195 (78.6) | 126 (77.3) |  |
| ***Vascular risk factors*** | | | |
| Hypertension | 148 (59.7) | 111 (68.1) | 0.084 |
| Diabetes mellitus | 40 (16.1) | 35 (21.5) | 0.170 |
| Prior ischaemic stroke | 29 (11.7) | 26 (16.0) | 0.215 |
| Drinking status |  |  | 0.288 |
| Current drinker | 40 (16.1) | 37 (22.7) |  |
| Former drinker | 4 (1.6) | 1 (0.6) |  |
| Never drinker | 191 (77.0) | 115 (70.6) |  |
| Unknown | 13 (5.2) | 10 (6.1) |  |
| ***Stroke severity and early function*** | | | |
| Initial NIHSS score | 2.56 ± 2.27 | 4.50 ± 2.35 | <0.001 |
| Admission 24-h mRS score | 1.74 ± 1.11 | 2.84 ± 0.96 | <0.001 |
| Initial GCS score | 15.00 [15.00, 15.00] | 15.00 [15.00, 15.00] | 0.510 |
| Limb weakness | 151 (60.9) | 128 (78.5) | <0.001 |
| Dysarthria | 64 (25.8) | 71 (43.6) | <0.001 |
| Unable to walk within 48 h | 10 (4.0) | 36 (22.1) | <0.001 |
| Admission to stroke unit | 141 (56.9) | 102 (62.6) | 0.248 |
| Initial systolic blood pressure, mmHg | 156.00 ± 28.03 | 157.86 ± 25.84 | 0.490 |
| Initial diastolic blood pressure, mmHg | 91.00 [79.00, 102.00] | 90.00 [79.50, 100.00] | 0.398 |
| Initial pulse rate, beats/min | 82.50 ± 13.77 | 81.88 ± 13.13 | 0.645 |
| Initial body temperature, °C | 36.50 [36.30, 36.50] | 36.50 [36.35, 36.60] | 0.019 |
| Onset-to-arrival time, h | 17.88 [4.60, 50.24] | 18.36 [7.25, 49.01] | 0.502 |
| ***White matter hyperintensity burden*** | | | |
| Modified Fazekas score | 1.88 ± 0.80 | 1.99 ± 0.81 | 0.157 |
| High Fazekas burden | 152 (61.3) | 110 (67.5) | 0.201 |
| ***Inflammatory-metabolic biomarkers*** | | | |
| White blood cell count | 7.08 [5.88, 8.62] | 7.35 [6.16, 8.48] | 0.211 |
| Neutrophil count | 4.30 [3.52, 5.67] | 4.92 [3.86, 5.82] | 0.034 |
| Lymphocyte count | 1.90 [1.43, 2.29] | 1.77 [1.37, 2.28] | 0.292 |
| Neutrophil-to-lymphocyte ratio | 2.33 [1.75, 3.39] | 2.77 [1.86, 3.82] | 0.025 |
| Fibrinogen | 2.90 [2.56, 3.29] | 2.96 [2.60, 3.43] | 0.324 |
| Fasting glucose | 5.16 [4.63, 5.88] | 5.39 [4.70, 6.77] | 0.191 |
| Glycated haemoglobin | 5.80 [5.60, 6.50] | 5.90 [5.50, 7.00] | 0.807 |
| LDL cholesterol | 3.09 ± 0.87 | 3.10 ± 0.88 | 0.905 |
| Serum uric acid | 377.47 ± 107.18 | 363.37 ± 94.90 | 0.166 |
| Serum creatinine | 76.60 [62.20, 90.40] | 73.85 [60.60, 86.67] | 0.214 |
| Blood urea nitrogen | 5.19 [4.28, 6.01] | 4.83 [4.02, 5.88] | 0.097 |
| Alanine aminotransferase | 20.00 [14.00, 28.00] | 20.00 [14.00, 33.00] | 0.796 |

Note. Values are presented as mean ± standard deviation, median [interquartile range], or n (%), as appropriate. P values were calculated using Student's t test, Mann-Whitney U test, chi-square test, or Fisher's exact test, as appropriate. NIHSS, National Institutes of Health Stroke Scale; mRS, modified Rankin Scale; GCS, Glasgow Coma Scale; LDL, low-density lipoprotein.

**Table S2. Candidate model performance for the exploratory discharge NIHSS score ≥ 3 outcome**

| **Model** | **Accuracy** | **F1 score** | **AUROC** | **Sensitivity** | **Specificity** | **AUPRC** | **Precision** | **NPV** | **Brier score** | **Selected model** |
| --- | --- | --- | --- | --- | --- | --- | --- | --- | --- | --- |
| Random forest | 0.710 | 0.232 | 0.818 | 0.135 | 0.986 | 0.683 | 0.818 | 0.704 | 0.182 | Yes |
| LightGBM | 0.742 | 0.535 | 0.811 | 0.459 | 0.878 | 0.664 | 0.642 | 0.772 | 0.161 | No |
| Elastic net logistic regression | 0.727 | 0.619 | 0.802 | 0.684 | 0.748 | 0.597 | 0.565 | 0.832 | 0.188 | No |
| LASSO logistic regression | 0.730 | 0.621 | 0.801 | 0.684 | 0.752 | 0.595 | 0.569 | 0.833 | 0.186 | No |
| Logistic regression | 0.742 | 0.500 | 0.787 | 0.398 | 0.906 | 0.620 | 0.671 | 0.759 | 0.173 | No |
| RPART | 0.730 | 0.528 | 0.772 | 0.466 | 0.856 | 0.587 | 0.608 | 0.770 | 0.179 | No |
| Kernel K-nearest neighbours | 0.727 | 0.404 | 0.739 | 0.286 | 0.939 | 0.582 | 0.691 | 0.733 | 0.184 | No |
| Naive Bayes | 0.436 | 0.506 | 0.668 | 0.895 | 0.216 | 0.473 | 0.353 | 0.811 | 0.544 | No |

Note. Performance was estimated using stratified 10-fold cross-validation. Hyperparameters were tuned within each training partition using inner 5-fold cross-validation. SMOTE was not used in the primary analysis. AUROC was used as the primary selection metric. AUROC, area under the receiver operating characteristic curve; AUPRC, area under the precision-recall curve; NPV, negative predictive value.

**Table S3. Incremental predictive value of modified Fazekas score and inflammatory-metabolic biomarkers across outcomes**

| **Outcome** | **Feature set** | **Model** | **AUROC** | **AUPRC** | **ΔAUROC** | **ΔAUPRC** |
| --- | --- | --- | --- | --- | --- | --- |
| Early rehabilitation need | Clinical variables only | Logistic regression | 0.682 | 0.591 | 0.000 | 0.000 |
| Early rehabilitation need | Clinical + modified Fazekas score | Logistic regression | 0.679 | 0.575 | -0.003 | -0.016 |
| Early rehabilitation need | Clinical + biomarkers | Logistic regression | 0.669 | 0.544 | -0.013 | -0.047 |
| Early rehabilitation need | Integrated model | Logistic regression | 0.670 | 0.544 | -0.012 | -0.047 |
| Discharge mRS ≥ 2 | Clinical variables only | Logistic regression | 0.753 | 0.667 | 0.000 | 0.000 |
| Discharge mRS ≥ 2 | Clinical + modified Fazekas score | Logistic regression | 0.752 | 0.670 | -0.001 | 0.003 |
| Discharge mRS ≥ 2 | Clinical + biomarkers | Logistic regression | 0.729 | 0.640 | -0.024 | -0.027 |
| Discharge mRS ≥ 2 | Integrated model | Logistic regression | 0.730 | 0.645 | -0.023 | -0.022 |
| Discharge NIHSS ≥ 3 | Clinical variables only | Logistic regression | 0.751 | 0.606 | 0.000 | 0.000 |
| Discharge NIHSS ≥ 3 | Clinical + modified Fazekas score | Logistic regression | 0.749 | 0.605 | -0.002 | -0.001 |
| Discharge NIHSS ≥ 3 | Clinical + biomarkers | Logistic regression | 0.732 | 0.583 | -0.019 | -0.023 |
| Discharge NIHSS ≥ 3 | Integrated model | Logistic regression | 0.730 | 0.581 | -0.021 | -0.025 |

Note. Delta values were calculated relative to the clinical-variable model within each outcome. Positive ΔAUROC and ΔAUPRC values indicate improved discrimination. Biomarkers refer to inflammatory-metabolic biomarkers. All models used logistic regression. AUROC, area under the receiver operating characteristic curve; AUPRC, area under the precision-recall curve.

**Table S4. Sensitivity analyses for the primary early rehabilitation need model**

| **Analysis** | **Selected model** | **AUROC** | **95% CI** | **AUPRC** | **Brier score** | **Sensitivity** | **Specificity** | **Calibration intercept** | **Calibration slope** |
| --- | --- | --- | --- | --- | --- | --- | --- | --- | --- |
| Primary no-SMOTE analysis | Logistic regression | 0.670 | 0.614-0.719 | 0.544 | 0.216 | 0.355 | 0.859 | -0.122 | 0.776 |
| SMOTE sensitivity analysis | Random forest | 0.671 | Not estimated | 0.576 | 0.226 | 0.490 | 0.707 | -0.304 | 1.984 |
| Complete-case analysis | Random forest | 0.650 | Not estimated | 0.577 | 0.219 | 0.230 | 0.945 | 0.172 | 1.392 |
| Excluding patients with pre-stroke mRS ≥ 2 | Logistic regression | 0.670 | Not estimated | 0.574 | 0.214 | 0.427 | 0.829 | -0.145 | 0.700 |
| Reduced non-functional model | Logistic regression | 0.590 | Not estimated | 0.441 | 0.238 | 0.277 | 0.828 | -0.322 | 0.365 |

Note. The primary no-SMOTE analysis was prespecified as the main analysis. SMOTE, complete-case analysis, exclusion of patients with pre-stroke disability, and the reduced non-functional model were performed as sensitivity analyses. The reduced non-functional model excluded direct neurological severity and early functional predictors. Confidence intervals were not estimated for sensitivity analyses. SMOTE, synthetic minority oversampling technique; AUROC, area under the receiver operating characteristic curve; AUPRC, area under the precision-recall curve; CI, confidence interval; mRS, modified Rankin Scale.
